# Cognate beginnings to bilingual lexical acquisition

**DOI:** 10.1111/cdev.14170

**Published:** 2024-09-23

**Authors:** Gonzalo Garcia‐Castro, Daniela S. Avila‐Varela, Ignacio Castillejo, Nuria Sebastian‐Galles

**Affiliations:** ^1^ Center for Brain and Cognition Universitat Pompeu Fabra Barcelona Spain; ^2^ Institute for the Future of Education Tecnológico de Monterrey Bilbao Spain; ^3^ Departamento de Psicología Universidad Autónoma de Madrid Madrid Spain

## Abstract

Recent studies suggest that cognateness boosts bilingual lexical acquisition. This study proposes an account in which language co‐activation accelerates accumulation of word‐learning instances across languages. This account predicts a larger cognate facilitation for words in the lower‐exposure language than in the higher‐exposure language, as the former receive co‐activation from their translations more frequently. Bayesian Item Response Theory was used to model acquisition trajectories for 604 Catalan‐Spanish translations from a dataset of 366 12–32 month‐old bilinguals (*M* = 22.23 months, 175 female, mainly White, collected 2020–2022). Results show a larger cognate facilitation for words in the lower‐exposure language (*d* = .276), than for words in the higher‐exposure language (*d* = .022), supporting a language exposure‐moderated account for the effect of cognateness on lexical acquisition.

**Abbreviations**
HDI: highest density interval
ROPE: region of practical equivalence

The foundations of word learning are in place at an early age. At 6 months, infants start directing their gaze to objects when hearing their labels (Bergelson & Swingley, 2012; Tincoff & Jusczyk, 1999), and shortly after caregivers start reporting some words as acquired by their infant in vocabulary checklists (e.g., Fenson et al., 2007). Most research on early word acquisition relies extensively on data from monolingual children and is oblivious to the fact that a substantial proportion of the world population acquires more than one language from an early age (Grosjean, 2021). Previous work on bilingual vocabulary acquisition pointed to bilingual toddlers knowing, on average, fewer words in each of their languages than their monolingual peers, and to both groups knowing a similar number of words—if not more words—when the bilinguals' two languages are pooled together. Hoff et al. (2012) found that English‐Spanish bilingual toddlers in South Florida (United States) knew fewer words in English than monolinguals did, but both groups knew a similar total amount of words when both English and Spanish vocabularies were counted together. Other studies have provided converging evidence that bilinguals know a similar or even larger number of words than monolinguals when the two languages are aggregated (Gonzalez‐Barrero et al., 2020; Pearson & Fernández, 1994). Recently, more detailed analyses of the words included in bilinguals' lexicons have shown insights into the underlying mechanisms.

One important observation of studies on bilinguals' early vocabulary acquisition is that cognate words are easier to acquire than non‐cognate words (e.g., Bosch & Ramon‐Casas, 2014; Mitchell et al., 2023; Schelletter, 2002). Cognate words are translation equivalents that are phonologically similar (or share some type of form‐similarity). For instance, the Spanish translation equivalent of *train* is *tren*, a cognate word; the translation equivalent of *dog* is *perro*, a non‐cognate word. Mind that, since phonological similarity is a *continuum*, so is cognateness. The labels ‘cognate’ and ‘non‐cognate’—widespread in the literature and used in the present work—describe two sides of the same dimension, as opposed to clear cut categories. For historical reasons, some pairs of languages share more cognates than others: typologically close languages (like Dutch and English or Italian and Spanish) share more cognates than typologically distant languages (like English and Chinese, or Urdu and Spanish). The conclusion that cognate words are easier to learn is based on two types of evidence: studies investigating vocabulary sizes in children learning language pairs with different percentages of cognates (that is, differing in their typological distance) and studies comparing the number of cognate and non‐cognate words children know in a specific language pair.

Floccia et al. (2018) published an impressive study comparing the vocabularies of children learning several language pairs differing in their percentage of cognates. The authors collected vocabulary data on word comprehension and production from 372 24‐month‐old bilingual toddlers living in the United Kingdom who were learning English and an additional language. The additional language was one of 13 typologically diverse languages: Bengali, Cantonese Chinese, Dutch, French, German, Greek, Hindi or Urdu, Italian, Mandarin Chinese, Polish, Portuguese, Spanish, and Welsh. The authors calculated the average lexical overlap between the words in each of these additional languages and their translation equivalents in English. Lexical overlap was calculated in terms of phonological similarity (described below) and it was taken as a proxy of the degree of cognateness between each pair of languages. Floccia and co‐workers reported an increase in vocabulary size in the additional language (i.e., not English) associated with an increase in the average phonological similarity between the translation equivalents of each language pair. For example, English‐Dutch bilinguals (languages with a high phonological overlap), were able to produce more Dutch words than English‐Mandarin bilinguals (languages with a low phonological overlap) were able to produce in Mandarin. Blom et al. (2020), Bosma et al. (2019), and Gampe et al. (2021) reported similar results, providing converging evidence of a facilitatory effect of a lower language distance (i.e., a higher degree of cognateness) on vocabulary size.

A second set of studies suggested that cognates are overrepresented in bilinguals' early lexicon. Bosch and Ramon‐Casas (2014) collected parental reports of expressive vocabulary from 48 Catalan‐Spanish bilinguals aged 18 months and found that cognates represented a larger proportion of vocabulary than non‐cognates. Schelletter (2002) provided converging evidence from a longitudinal single‐case study, in which an English‐German bilingual child produced cognates earlier than non‐cognates, on average. But the high proportion of cognates in the vocabulary of the participants in these two studies may not necessarily be evidence of a facilitation effect of cognateness, but rather of simply the high proportion of cognates present in the pair of languages being learned. For instance, if two given languages share a high proportion of cognates like 70%, the vocabulary contents of children learning both languages should, in principle, approximate such proportion of cognates, even in the absence of a cognateness facilitation effect. More recently, Mitchell et al. (2023) addressed this issue in a longitudinal study. The authors collected expressive vocabulary data of 47 16‐ to 30‐month‐old French‐English bilinguals living in Canada, in both languages. They created two lists of translation equivalents; one made of 131 cognates, and one made of 406 non‐cognates. Across ages, the proportion of words that children were reportedly able to produce was higher in the cognate lists than in the non‐cognate list. Critically, this difference persisted after both lists were matched in size, controlling their semantic category (i.e., furniture, animals, food were similarity represented in both lists) and age‐of‐acquisition norms (an index of word difficulty). Taken together, the results of these two lines of research support the hypothesis that phonological similarity (as reflected in cognateness) plays a facilitation role in bilingual word learning.

Parallel activation of bilinguals' lexicons has been proposed as the underlying mechanism for such facilitatory effect (e.g., Floccia et al., 2018; Mitchell et al., 2023). The parallel activation hypothesis stems from the language non‐selective account of lexical access, which suggests that bilinguals activate both languages simultaneously during language processing, even in fully monolingual contexts. Evidence with adult bilinguals supporting the language‐non selective account of lexical access has been reported for language comprehension and production, across the auditory and visual (reading and signing) modalities (e.g., Hoshino & Kroll, 2008; Shook & Marian, 2012; Spivey & Marian, 1999). One of the clearest pieces of evidence of parallel activation was provided by Costa et al. (2000). In this study, Spanish monolinguals and Catalan‐Spanish bilingual adults were asked to name pictures of common objects in Spanish. In half of the trials, the object labels were cognates in Spanish and Catalan (*árbol*‐*arbre*, translations of *tree*), whereas in the other half of the trial labels were non‐cognates (*mesa*‐*taula*, translations of *table*). Obviously, such distinction was only relevant for bilinguals. Bilinguals named cognate pictures faster than non‐cognate pictures, even after adjusting for the lexical frequency of the items. In contrast, Spanish monolinguals, who were unfamiliar with the Catalan translations of the Spanish words they uttered, showed equivalent naming times for the two types of stimuli. The authors interpreted the difference between cognates and non‐cognates in bilinguals as reflecting the additional phonological activation that cognate words would receive from their translation equivalents (due to language non‐selective activation of bilinguals' lexicons). These results showed that bilinguals' Catalan phonology was activated during the production of Spanish words, facilitating the naming of cognate pictures. More recently, evidence of parallel activation has been reported in bilingual toddlers and children too (Bosma & Nota, 2020; Floccia et al., 2020; Von Holzen et al., 2019). Although there is a consensus on the role of parallel activation in bilinguals' lexical processing and acquisition, previous studies do not address its influence on the learning trajectories of individual words. Results are aggregated across words and provide no information about the specific dynamics of how parallel activation influences word learning. This is the goal of the present research.

We propose an account in which a learning instance for a word may also represent a learning instance for its translation equivalent, to the extent that such translation equivalent is co‐activated. We use the term *learning instance* in the fashion of accumulator models of language acquisition: exposure to a word form that constitutes an opportunity for the child to accumulate information about the word (Kachergis et al., 2022; Mollica & Piantadosi, 2017). We do not assume if a learning instance is a discrete or a continuous unit of accumulation of information. We consider that a word exposure may contribute a word‐learning instance in a degree proportional to the strength of the activation of its lexical representation. This activation may result from the infant being exposed to the actual word form, or the result of activation spreading through phonological or semantic links across lexical representations, as in the case of parallel activation. The strength of this co‐activation is proportional to the phonological similarity between the two translation equivalents; given that cognates share higher phonological similarity than non‐cognates, the former should be co‐activated more strongly than the latter. This should lead to a faster accumulation of learning instances for cognates, compared to non‐cognates. Parallel activation would allow bilingual children to accumulate learning instances for words in both languages even during fully monolingual situations, but the impact of this mechanism would be asymmetric across languages: words from the lower‐exposure language would receive stronger activation from words in the higher‐exposure language than vice versa. Another mechanism that might result in an asymmetric effect of cognateness is the fact that the acquisition trajectories of words in the higher‐exposure language might reach a ceiling before those of words in the lower‐exposure language. As words in the higher‐exposure language are acquired at earlier ages, it is the acquisition of words in the lower‐exposure language that are left to benefit from the cognateness facilitation effect. In summary, we hypothesize that the acquisition of words from the lower‐exposure language (or non‐dominant language) would benefit more strongly from their cognate status than the acquisition of words from the higher‐exposure language (or dominant language). This asymmetric cross‐language activation would be consistent with previous reports of larger priming effects from the dominant to the non‐dominant language (e.g., Grainger, 1998).

Consider the example of the Catalan‐Spanish cognate translation equivalent /ˈgat/−/ˈga.to/ [*cat*], which are phonologically very similar. When the child listens to /ˈgat/, they will strongly co‐activate /ˈga.to/ in parallel. If the child has already formed a form‐meaning association for both word forms, parallel activation may result from the activation of their common concept or from activation spreading throughout phonological similarity. We assume semantic co‐activation to be constant across cognate and non‐cognate translation equivalents, and focus on phonological co‐activation as an additional source of activation that affects cognates more strongly than non‐cognates. Therefore, this exposure will count as a learning instance for both co‐activated forms. The case of the non‐cognate translation equivalent /ˈgos/−/ˈpe.ro/ [*dog*] would be different. Given the low phonological similarity between both word forms, an exposure to /ˈgos/ will result in a weak activation of /ˈpe.ro/, leading to such exposure counting as a learning instance for /ˈgos/ (which the child was exposed to), but not for /ˈpe.ro/. While /ˈgat/−/ˈga.to/ will benefit from phonological co‐activation, /ˈgos/−/ˈpe.ro/ will not. If the child receives linguistic input from one of the languages more often than from the other, this effect might affect each form of the cognate translation equivalent differently. For instance, if the child receives a larger amount of Catalan input than Spanish input, they will encounter the Catalan form /ˈgat/ more frequently than the Spanish form /ˈga.to/. Through parallel activation, /ˈgat/ will activate /ˈga.to/ more often than vice versa. Ultimately, /ˈga.to/ will benefit more strongly from its cognate status than /ˈgat/, as it receives additional learning instances from its translation equivalent more often than /ˈgat/.

To test these predictions, we created a vocabulary questionnaire and collected vocabulary data on production and comprehension from a large sample of bilingual children living in the Metropolitan Area of Barcelona (Catalonia, Spain), and who were learning to Catalan and Spanish. Catalan and Spanish are two Romance languages that co‐exist in Catalonia in a co‐official status. Both languages are used in fairly similar contexts, and both are known by the majority of the population. In 2018, more than 81.2% of a representative sample of 8780 adults aged 15 years or older living in Catalonia reported being able to speak Catalan, and more than 99.5% of the same population reported being able to speak Spanish (Generalitat de Catalunya, 2018). Together with the fact that Catalan and Spanish share a considerable amount of cognates, this makes Catalan‐Spanish bilingual children an ideal population in which to investigate the role of cognateness of vocabulary acquisition.

We adopted a Bayesian explanatory item response theory approach to model the probability of acquisition of 604 Catalan and Spanish nouns included in the vocabulary checklist. Words were considered as acquired if caregivers reported such words to be understood (comprehension) or understood and said (production) by their child. We estimated the impact of several predictors of interest on the probability of acquisition, including participants' age, participants' degree of exposure to each language, and the cognate status of the word forms. We also adjusted the estimated effects for the other two known to contribute to word acquisition, which we modeled as covariates: word‐form length and lexical frequency. We predicted an interaction between the cognate status of word forms and participants' degree of exposure to the language each word form belongs to. We anticipated this interaction to be revealed by a higher probability of acquisition of cognate words in the lower‐exposure language, but not of cognate words in the higher‐exposure language.

## METHODS

Data and materials for this research are available on the Open Science Framework (https://osf.io/hy984), and code is available on GitHub (https://github.com/gongcastro/cognate‐beginnings). For reproducibility, a Docker image of the RStudio session is available on DockerHub (https://hub.docker.com/repository/docker/gongcastro/cognate‐beginnings/general).

### Participants

We collected 436 responses to the vocabulary questionnaire, corresponding to 366 distinct children (175 female, 179 male, 12 not reported, mainly White). Participants were aged 12–32 months (*M* = 22.23, SD = 4.88, range = 12.06–31.93) (Table 1). Of those participants, four participated four times, eight participated three times, 42 participated twice, and 312 participated once. Recurrent participants provided responses with a minimum of 25 days between responses, and a maximum of 527 days.

**TABLE 1:** Participant sample size by age and degree of exposure to Catalan.

Catalan exposure	Age (months)					
	[10–14]	(14, 18]	(18, 22]	(22–26]	(26–30]	(30–34]
75%–100%	18	23	36	38	20	7
50%–75%	8	13	30	41	18	1
25%–50%	10	17	45	29	17	0
0%–25%	7	11	21	17	8	1
*N*	43	64	132	125	63	9

Participants were residents in the Metropolitan Area of Barcelona (Catalonia, Spain). Data collection took place between March 30th, 2020 and October 31th, 2022. Participants were part of the database of the Laboratori de Recerca en Infància from Universitat Pompeu Fabra and were contacted by e‐mail or phone if their child was aged between 12 and 32 months. Upon consent, families were sent a link to the questionnaire via e‐mail, which they filled from a computer, laptop, or mobile device. Filling the questionnaire took 30 min approximately. After completion, families were rewarded with a token of appreciation. Before filling out the vocabulary questionnaire, parents completed a brief demographic survey and a language exposure questionnaire. The demographic survey collection information about the childs' sex and caregivers' educational attainments.

We used the highest self‐reported educational attainment of parents or caregivers as a proxy of participants' socio‐economic status. This information was provided by each parent or caregiver by selecting one of six possible alternatives in line with the current educational system in Spain: *sense escolaritzar/sin escolarizar* [no education], *educació primària/educación primaria* [primary school], *educació secundària/educación secundaria* [secondary school], *batxillerat/bachillerato* [complementary studies/high school], *cicles formatius/ciclos formativos* [vocational training], and *educació universitària/educación universitaria* [university degree]. Most families reported university studies (356, 82%), followed by families where the highest educational attainment were vocational studies (59, 14%), secondary education (8, 2%), complementary studies (6, 1%), primary education (1, <1%), and no formal education (2, <1%).

The language exposure questionnaire presented caregivers with an extensive list of languages. Caregivers were instructed to select those languages to which the child was exposed to on a regular basis at any point in their lives, regardless of the source of such input. Caregivers were then asked to provide an estimated proportion of exposure that their child had accumulated from each of the selected languages from birth to the day in which they answered the questionnaire (e.g, 60% Catalan, 32% Spanish, 8% Arabic). For each participant included in the sample, we gathered two language exposure data points: one for Catalan and one for Spanish. Participants exposed to a language other than Catalan or Spanish in more than 10% were excluded from further analyses. The final dataset comprised 70 participants (16.06%) who were exposed to a third language, but whose exposure to such language was less than 10%. All families provided informed consent before participating.

### Questionnaire

To collect vocabulary data from participants, we created an ad hoc questionnaire. This questionnaire was inspired by the MacArthur‐Bates Communicative Development Inventory (Fenson et al., 2007) and its adaptations to other languages, and was implemented on‐line using the formr platform (Arslan et al., 2020). This questionnaire is structured in three blocks: (1) a language exposure questionnaire, (2) a demographic survey, and (3) two vocabulary checklists. Vocabulary checklists consisted of two lists of words, one in Catalan and one in Spanish. Both lists included items from a diverse sample of 26 semantic or functional categories (see Table 2). The Catalan checklist contained 793 items and the Spanish checklist contained 797. Items in one language were translation equivalents of the items in the other language (e.g., the same participant responded to both *gos* and *perro*, Catalan and Spanish for *dog*), roughly following a one‐to‐one mapping. Some of the words in Catalan did not have a clear translation or had more than one possible translation in Spanish, and vice versa, therefore the unequal number of words included in the two lists. Before starting to fill out each of the vocabulary checklists, caregivers were instructed to not rely on their responses to words in one language when providing responses to words in the other language (e.g., if the child understands the Spanish word *barco* [*boat*], the child may not necessarily be able to understand its Catalan translation *vaixell*).

**TABLE 2:** Summary of the translation equivalents included in the final analyses.

Semantic category	List A	List B	List C	List D	Examples
Household items	31	26	30	25	Clock, video
Food and drink	29	26	23	27	Sausage, yogurt
Animals	26	23	19	25	Panther, tiger
Outside	14	13	13	15	Farm, stone
Body parts	14	12	11	11	Face, finger
Toys	11	11	12	13	Piano, racket
Clothes	12	12	10	10	Zipper, sandal
Vehicles	9	10	11	10	Helicopter, tractor
Colors	6	6	6	6	Red, green
People	7	4	6	6	Police, babysitter
Furniture and rooms	4	4	4	4	Corridor, terrace
Time	2	2	2	2	Day, night
Adventures	1	1	1	1	Witch
Parts of things	1	1	1	1	Wheel

For each word included in the vocabulary checklists, we asked parents or caregivers to report whether their child was able to understand it, understand *and* say it, or did not understand or say it (checked out by default). Given the large number of words in the vocabulary checklists, we created four different subsets of the complete list of items (A, B, C, and D). Each subset contained a random but representative sub‐sample of the items from the complete list (see Table 2). Semantic or functional categories with less than 16 items—thus resulting in less than four items after dividing it in four subsets—were not divided; all of their items were included in the four subsets Items that were part of the trial lists of some ongoing experiments in the lab were also included in all versions. The resulting reduced list contained between 343 and 349 Catalan words, and between 349 and 371 Spanish words. Participants were randomly allocated into one of the four questionnaire subsets (A, B, C, or D). Each participant was always allocated to the same subset. All responses—longitudinal or not—were passed to the data processing pipeline.

To compute predictors of interest, we manually generated a broad phonological transcription of every word included in the vocabulary checklists in X‐SAMPA format. Catalan word forms were transcribed to Central Catalan phonology, and Spanish word forms were transcribed to Castilian Spanish phonology.

### Data analysis

#### Data processing

We collected data for 1590 words. We restricted the analyses to responses to nouns (628 items corresponding to other grammatical classes were excluded). We then excluded items with missing lexical frequency scores (*n* = 269, see Model predictors section), items that included more than one lemma (e.g., *mono/mico* [monkey], *n* = 48), multi‐word items or phrases (e.g., *barrita de cereales* [cereal bar], *n* = 9). Finally, we removed items without a translation in the other language (*n* = 32). This resulted in a final list of 604 items, corresponding to 302 Catalan words and their 302 Spanish translations (302 translation equivalents). After collecting participants' responses, the final dataset consisted of 138,078 observations, each corresponding to a single response of one participant to one item. Each translation equivalent received a median of 234 responses (Min = 106, Max = 872) from participants, both languages pooled together. Data processing and visualization was done in R (R Core Team, 2013, version 4.3.3).

#### Modeling approach

We modeled the probability of participants answering each response category (*No* < *Understands* < *Understands and Says*) using a Bayesian, multilevel, ordinal regression model. This model allowed us to estimate the effect of our predictors of interest on participants' probability of word‐acquisition for comprehension and production simultaneously. Previous studies have modeled comprehension and production separately, also using separate samples of participants of different ages, collecting comprehension data in younger infants, and production data in older infants (Braginsky et al., 2019; e.g., Fenson et al., 2007; Floccia et al., 2018). We did not have clear predictions about whether cognateness would facilitate word acquisition more strongly in the comprehension or the production modality. For this reason, we took advantage of the features of ordinal models to model both modalities together in the sample, therefore providing a more extensive insight into the effect of cognateness across a wider range of ages (see Bürkner & Vuorre, 2019 for a more extensive description of the advantages and implementation of Bayesian ordinal regression models).

Our predictors of interest were *Age* (number of months elapsed between participants' birth date and questionnaire completion), *Length* (number of phonemes in the X‐SAMPA phonological transcription of the word form), *Frequency* (lexical frequency of the word form), *Exposure* (degree of exposure to the language the word form belongs to), and *Cognateness* (defined as the phonological similarity between translation equivalents). A more detailed description of these predictors is provided in the Model Predictors section. We added these variables as main effects, together with the two‐way and three‐way interactions between *Age*, *Exposure*, and *Cognateness*. We included per‐participant random intercepts and random slopes for *Age*, *Exposure*, *Cognateness*, and their two‐ and three‐way interactions. We included per‐translation equivalent random intercepts and random slopes for *Age* and *Exposure*, and their two‐way interaction. The model also estimated the standard deviation of every random effect and all correlation parameters between random effects. This maximal random effects structure allowed us to account for the potential correlation between observations from the same participant (within and across questionnaire administrations), and observations from the same item. We specified a weakly informative prior around the parameters of the model. We used a $N\left(-0.\;25,0.\;50\right)$ prior for the fixed intercepts, a $N\left(0,1\right)$ prior for the fixed slopes, a $N_{+}\left(1,0.\;25\right)$ prior for the standard deviation of the random effects, and a $\text{LKJCorr}\left(2\right)$ prior for the correlation parameters. See Section S2 for a detailed description of the model and its diagnostics.

We implemented the model using brms (Bürkner, 2017), an R interface to the Stan probabilistic language (2.32.1) (Carpenter et al., 2017). We ran four iteration chains using the by‐default No U‐Turn Sampler algorithm with 1000 iterations each and an additional 1000 warm‐up iterations per chain.

#### Model predictors

Lexical frequencies were extracted from the CHILDES database (MacWhinney, 2000). Due to the low number of Catalan participants and of Catalan tokens available in their corresponding CHILDES corpora (see Section S1), using lexical frequencies from Catalan and Spanish was not possible. Instead, we extracted lexical frequencies from the CHILDES English corpora. We mapped the lexical frequencies of the English words to their Catalan and Spanish translations (see Braginsky et al., 2019; Fourtassi et al., 2020; for a similar approach). We transformed the resulting lexical frequencies to Zipf scores.

We estimated participants' degree of exposure to each language (*Exposure* predictor) using caregivers' responses to the language exposure questionnaire that they filled in before the vocabulary checklists, in which caregivers provided an estimated proportion of the child's exposure to each language (Catalan, Spanish, or any other). We assigned the Catalan and Spanish scores to participants' responses to Catalan and Spanish word forms, respectively. For instance, for a participant reported to be exposed 65% of the time to Catalan, their associated responses to Catalan word forms (e.g., *taula*, *gos*) would be assigned 0.65, their responses to Spanish word forms (e.g., *mesa*, *perro*) would be assigned 0.35.

Following Floccia et al., we defined *Cognateness* as the phonological similarity between each word form and its translation. For each translation equivalent, we calculated the Levenshtein distance between the Catalan and the Spanish phonological transcriptions of the word forms. The Levenshtein distance measures the number of editions (insertions, deletions, or substitutions) that one string of characters must go through to become identical to the other. We divided the Levenshtein distance of each translation equivalent by the length of the longest word form to correct for word‐form length (longer strings are likely to show a larger number of mismatches). Finally, we subtracted the result from one so that it could be interpreted in terms of phonological similarity, instead of phonological distance. This led to a distance metric that ranged from zero to one, where zero indicates that both word forms are completely different (e.g., /ˈtaw.lə/−/ˈme.sa/, *table*), and one indicates that the two word forms are identical (e.g., /ˈmar/−/ˈmar/, *sea*). Predictors were standardized before entering the model by subtracting the mean of the predictor from each value and dividing the result by the standard deviation of the predictor.

#### Statistical inference

We assessed the practical relevance of the estimated regression coefficients of the model following Kruschke and Liddell (2018). First, we specified a region of practical equivalence (ROPE) from −0.025 to +0.025, in the probability scale. This region indicates the range of values that we considered equivalent to zero. We then summarized the posterior distribution of each regression coefficient with the 95% highest density interval (HDI). This interval contains the true value of this coefficient with 95% probability, given the data. Finally, we calculated the proportion of posterior samples in the 95% HDI that fell into the ROPE, noted as *p*(ROPE), which indicates the probability that the true value of the regression coefficient falls into the ROPE (and therefore should be considered equivalent to zero). For example, *p*(ROPE) = .80 indicates that, given our data, there is an 80% probability that the true value of the coefficient falls within the ROPE, and can therefore be considered equivalent to zero. See Section S2 for considerations about statistical power and sample size.

## RESULTS

**FIGURE 1:**
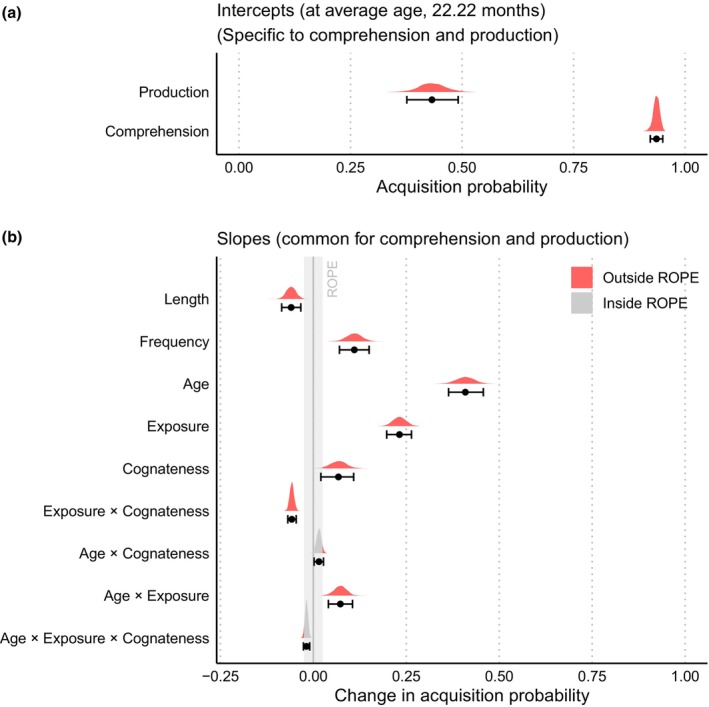
Posterior distribution of fixed regression coefficients. Values have been transformed to the probability scale for interpretability. The gray rectangle shows the region of practical equivalence (ROPE, [−0.025, +0.025]). Areas of the posterior distributions that overlap with the ROPE are shaded in gray. (a) Posterior distribution of intercepts for Comprehension and for Comprehension and Production. The *X*‐axis shows the estimated average probability of acquisition when all predictors are set at zero. These estimations correspond to the probability of comprehension and production for average values of all predictors (e.g., when age equals 22 months). (b) Posterior distribution of slopes. The *X*‐axis shows the estimated average change in probability of acquisition associated with a change of one standard deviation in the predictor, when the other predictors are set at zero.

We now present our confirmatory analysis and report a summary of the regression coefficients of interest. Figure 1a shows the posterior distribution of the intercept for *Comprehension* and *Production*, and Figure 1b shows the posterior distribution of the fixed regression coefficients, and their degree of overlap with the ROPE. For interpretability, we report the estimated regression coefficients transformed to the probability scale. The resulting values correspond to the maximum difference in probability of acquisition (*Comprehension* or *Comprehension and Production*) equivalent to a one standard deviation change in each predictor. Whenever possible, we also report the difference in probability of acquisition associated with a meaningful increase of the predictor (e.g., a 1‐month increment in age, or a one phoneme increment in word length). This value was calculated by dividing the median of the posterior of the coefficient by the standard deviation of the unstandardized variable.

As expected, the probability of acquisition decreased as word length increased (*β* = −.06, 95% HDI = [−.085, −.034], *p*(ROPE) = 0). For every additional phoneme, the probability of acquisition decreased to −.06. Lexical frequency was associated with an increase in the probability of acquisition (*β* = .11, 95% HDI = [.07, .15], *p*(ROPE) = 0). The coefficient of age showed the strongest association with the probability of acquisition (*β* = .409, 95% HDI = [.364, .457], *p*(ROPE) = 0). A 1‐month increment in age increased a maximum of .08 the probability of acquisition. The degree of language exposure (*Exposure*) had the second strongest effect on the probability of acquisition (*β* = .232, 95% HDI = [.197, .264], *p*(ROPE) = 0). The impact of this predictor on the probability of acquisition was positive: for every standard deviation increase in exposure, of a word being acquired in that language increased by .772 probability points. Around 5.35% of the posterior samples of the main effect of *Cognateness* overlapped with the ROPE (*β* = .068, 95% HDI = [.02, .109]). For every 10% increment in cognateness, the probability of word acquisition increased by .007.

The 95% HDI of the regression coefficient of the *Age* × *Exposure* interaction was positive (*β* = .073, 95% HDI = [.041, .105], *p*(ROPE) = 0), showing that the effect of the degree of language exposure on the probability of word acquisition differed across ages: older children were more likely to acquire words from the higher‐exposure language, compared to younger children. The 95% HDI of the regression coefficient of the *Age* × *Cognateness* interaction was positive, but overlapped with the ROPE almost completely (*β* = .015, 95% HDI = [.002, .027], *p*(ROPE) = .904), suggesting that the effect of the cognateness remained constant across ages.

**FIGURE 2:**
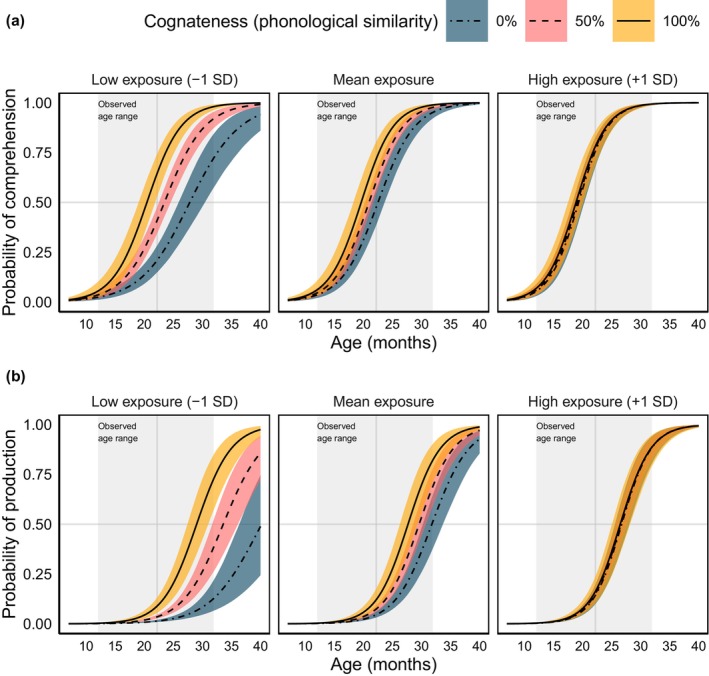
Posterior marginal effects for comprehension (a) and production (b). Lines and error bands correspond to the mean and 95% credible interval of the posterior‐predicted means. Different colors indicate different levels of cognateness (phonological similarity). Predictions are presented separately for different degrees of language exposure: Low exposure (−1 SD), mean exposure, and high exposure (+1 SD). In‐sample predictions lie inside the gray rectangles. For reference, we indicate the 50% chance level of word acquisition (horizontal gray lines), and the mean age of the sample (vertical gray lines).

The effect of *Cognateness* interacted with that of *Exposure*: the 95% HDI of the regression coefficient of interaction excluded the ROPE entirely (*β* = −.058, 95% HDI = [−.069, −.046], *p*(ROPE) = 0), suggesting that the effect of cognateness on a word's probability of acquisition changed depending on participants' exposure to the language the word belonged to. When a word form belonged to the lower‐exposure language (e.g., −1 SD exposure, or 0.79), *Cognateness* increased the probability of acquisition substantially, while this effect was negligible for words belonging to a higher‐exposure language (e.g., +1 SD exposure, or 0.19) (see Figure 2).

Finally, the coefficient of the three‐way interaction between *Age*, *Exposure*, and *Cognateness* was negative, but overlapped significantly with the ROPE (*β* = −.018, 95% HDI = [−.027, −.01], *p*(ROPE) = .903). This suggests that the effect of *Cognateness* on the probability of acquisition remained constant across the different combinations of *Age* and *Exposure* scores.

To rule out the possibility that cognateness facilitation effect we found was due to cognateness comprising more frequent syllables than non‐cognates—and therefore not because of their cognate status itself—we run an exploratory analysis comparing the syllabic frequency of cognates and non‐cognates included in our analyses. To calculate syllable frequency, we first extracted all syllables embedded in the selected words. For each syllable, we summed the lexical frequency of all the words in which such syllable appeared (we used the same frequencies as described in the Methods section). The resulting value provided an estimate of the number of times the syllable appears in child‐directed speech, embedded within different words. Finally, for each word form, we summed the frequency of its syllables, as an estimate of the syllabic frequency of the word form. We fit a Bayesian model with *Cognateness* as response variable, and the main effects of syllable frequency and number of syllables (to control for the fact words with more syllables are more likely to score higher in syllabic frequency) as predictors. This exploratory analysis provided strong evidence for the association between cognateness and syllabic frequency being equivalent to zero (see Section S3).

## DISCUSSION

This study investigated the impact of cognateness (i.e., phonological similarity between translation equivalents) on the early bilingual lexicon. We used Bayesian item response theory to model the acquisition trajectories of a large sample of Catalan and Spanish words, estimating the effect of cognateness on the probability of acquisition. This model corrected for word‐form length (number of phonemes) and lexical frequency, and participants' age and degree of language exposure. Overall, we found that cognates (i.e., phonologically similar translation equivalents) were acquired earlier than non‐cognates. This effect was moderated by the degree of language exposure. Word forms from the lower‐exposure language benefited from their cognate status, whereas word forms from the higher‐exposure language did not. Using the concept of the accumulator (see Kachergis et al., 2022 for review), we provide a theoretical account of bilingual lexical acquisition. In the present account, parallel activation of the two languages plays a central role during the acquisition of early representations in the bilingual lexicon, and in which the dynamics of co‐activation between translation equivalents result in an earlier age‐of‐acquisition.

The present investigation is particularly relevant in the light of two previous findings. First, Floccia et al. (2018) reported that bilingual toddlers learning two typologically close languages (e.g. shared many cognates, like English‐Dutch) showed larger vocabulary sizes than those learning typologically distant languages (e.g., shared fewer cognates, like English‐Mandarin). Second, Mitchell et al. (2023) found an earlier age‐of‐acquisition for cognates, compared to non‐cognates. The outcomes of both studies pointed to cognateness facilitating word acquisition through parallel activation, but the underpinnings of such effect were unclear. While parallel activation has been extensively described in experimental studies, current paradigms of bilingual word acquisition and word learning are, to a large extent, dissociated from the mechanisms proposed by previous work on word processing. The notion of *accumulator*, as conceptualized by accumulator models of language acquisition, may provide a convenient theoretical framework to narrow this gap.

Accumulator models devise word acquisition as a continuous process in which the child gathers information about words by accumulating learning instances with such words. When the number of cumulative learning instances for a word reaches some theoretical threshold, the child is considered to have acquired such a word. The rate at which a child accumulates learning instances with a word is a function of child‐level properties (e.g., ability, amount of quantitative language exposure) and word‐level properties (e.g., lexical frequency) (Hidaka, 2013). Through statistical inference, formalized accumulator models provide meaningful information about parameters of interest like the aforementioned predictors (Kachergis et al., 2022; Mollica & Piantadosi, 2017), and allow to generate quantitative predictions about age‐of‐acquisition and vocabulary growth under competing theoretical accounts (Hidaka, 2013; McMurray, 2007). Using the notion of accumulator, we extended this type of account to the bilingual case. We suggested that the cognate facilitation effect on bilingual word acquisition is the result of cognate words being activated more strongly by their translation than non‐cognates. This would lead cognate words to accumulate learning instances at a faster rate than non‐cognate words. When a bilingual child is exposed to a word form, they activate not only its corresponding lexical representation but also the lexical representation of its translation. The amount of co‐activation that spreads from the spoken word form to its translation is proportional to the amount of phonological similarity between both word forms. Cognates would receive more activation from their translation than non‐cognates, leading children to accumulate learning instances with cognate words at a faster rate than with non‐cognate words. As a result, lexical representations of cognate words would consolidate at earlier ages than those of non‐cognate words.

These predictions address a critical subject in bilingualism research. Do bilingual infants accumulate learning experiences in both languages independently, or does exposure to one language impact the acquisition trajectory of the other language? In the context of lexical acquisition, the former scenario predicts that every learning instance for a given word form contributes to the acquisition of the representation of such word in the lexicon, while the acquisition of its translation remains unaffected by such experience. In the latter scenario, a learning instance to the same word form would contribute not only to the acquisition of the representation of the word, but also, to some extent, to the acquisition of its translation. Our findings provide strong support for an account of bilingual vocabulary growth in which the experience and learning outcomes accumulated by the child in one language impact those in the other language through cross‐language phonological associations. Such a facilitatory mechanism might be an important piece in the puzzle of bilingual language acquisition. In particular, it may shed some light on why bilingual infants do not show relevant delays in language acquisition milestones (e.g., language discrimination, word‐recognition, vocabulary size), compared to their monolingual peers, while receiving a reduced quantity of speech input in each of their languages (see Sebastian‐Galles & Santolin, 2020 for review). In line with previous observations, infants in the present study benefited more strongly from the cognateness facilitation effect when acquiring words from the language of lower exposure than in the language of higher exposure.

This mechanism might be extended to provide a plausible explanation for the language similarity facilitation reported by Floccia et al. The authors observed a facilitation in the additional (non‐English) language. Children learning two typologically close languages knew more words in the additional language than those learning two typologically more distant languages. In their sample, the additional language was consistently also the lower‐exposure language for most children, while English was the higher‐exposure language. Given that words in English were more likely to be acquired first, higher phonological overlap for words in the language of lower exposure (especially those of lower lexical frequency) would facilitate vocabulary growth for languages sharing more cognates with English.

The asymmetric facilitation of cognateness on word acquisition reported in the present study parallels previous findings in toddlers and adults. For instance, unbalanced (or low‐proficiency) bilinguals benefit from cross‐language forward priming (dominant to non‐dominant) during word processing (Grainger, 1998; Singh, 2014; Von Holzen et al., 2019). One the other hand, backward priming (non‐dominant to dominant) seems less robust and more challenging to detect (e.g., Hoshino et al., 2010). Balanced (or high‐proficiency bilinguals) show an equivalent priming facilitation in both directions (Duñabeitia et al., 2009). These results have been taken as evidence for an asymmetry in the strength of forward and backward connections in the unbalanced bilingual lexicon. Although implemented in different ways, or found under different assumptions, such a dominance‐moderated asymmetry is accounted for by multiple models of lexical processing like the Revised Hierarchical Model (Kroll & Stewart, 1994), BIA/BIA+ (Dijkstra & Van Heuven, 2013), BLINCS (Shook & Marian, 2013), or Multilink (Dijkstra et al., 2019), and also by models providing a more development‐oriented perspective, like the Ontogenic Model (Bordag et al., 2022), and BIA‐d (Grainger et al., 2010). Overall, this provides an apparently convenient account for the interaction between language dominance and cognateness found in the present study. These models are aimed at explaining results in adults, and their predictions should be taken with caution when extended to early language acquisition.

In adult bilingual populations, language dominance and proficiency are frequently defined using dimensions other than degree of exposure, which is a more common practice in infant research (Rocha‐Hidalgo & Barr, 2023). For instance, low‐proficiency bilinguals in many of the aforementioned studies acquired their second language years after their toddlerhood. We identify three critical ways in which this prevents a clear comparison between our results and those from studies on second language acquisition in adults. First, in adult second language acquisition, the acquisition of the phonology of the new language must be negotiated with the already acquired phoneme inventory of the first language (e.g., Cutler et al., 2006), in place around the first year of life (see Werker & Hensch, 2015 for review). Second, adults acquiring a second language already possess a system of form‐meaning mappings, whereas simultaneous bilingual infants must build a lexicon for two languages in the absence of clear form‐meaning mappings. Third, adults are assumed to be literate and to possess an orthographic system in place, which may shape how new words are integrated in the lexicon and processed during experimental tasks (e.g., Thierry & Wu, 2007). In this scenario, the acquisition of a second language may take place in a substantially different way compared to how bilingual infants acquire two languages from birth. A more similar case to the one concerning the present study is considered by the DevLex‐II model (Zhao & Li, 2010), which captures unique features of the early bilingual lexicon, and considers the case of infants simultaneously acquiring their two languages. In line with the adult models, DevLex‐II predicts asymmetries between word representation from the dominant and the non‐dominant language. Simulations from DevLex‐II result in asymmetric cross‐language priming, in which words from the dominant (earlier acquired) language primed more strongly the recognition of words in the non‐dominant language (later acquired) than in the other direction (Zhao & Li, 2013).

In summary, there is a compelling case for attributing asymmetric effects of parallel activation to differences in activation strength between forward and backward connections. It is nonetheless possible that, as argued in the introduction, the asymmetric effect of cognateness found in the present study is simply the result of infants being exposed more frequently to words in the dominant language than to words in the non‐dominant language. This would lead to words in the non‐dominant language receiving additional parallel activation, compared to words in the dominant language, and therefore benefiting more strongly from their cognate status. These two accounts are not mutually exclusive, as words in the dominant language may activate more *strongly* their translations than vice versa, on top of such activation being more frequent. Further research is needed in order to clarify the role of the interplay between quantitative language exposure and lexical frequency on the facilitatory effect of cognateness.

The amount of times a bilingual child encounters a word form is a function of its lexical frequency in the corresponding language (i.e., how often a word form appears in the child's linguistic input in that language), and child's degree of exposure to that language (i.e., how often the child is exposed to that language, as opposed to other languages). In the present study, we modeled the role of the latter predictor, revealing its strong moderating role in the cognateness facilitation effect, while including lexical frequency as a co‐variate, in order to increase the accuracy of the model's predictions. We suggest that, in future studies, lexical frequency scores and degree of language exposure estimates be combined in the same model, either as a composite measure (i.e., a language exposure‐weighted lexical frequency score) or in interaction with other predictors of interest (e.g., in interaction with age and cognateness). An example of the former is provided in Section S4, in which we show the estimates of a model that includes a composite measure of lexical frequency and degree of language exposure. The estimations of this model are equivalent to those of the model in the main text. This is expected, as lexical frequencies were held constant across members of the same translation equivalents (i.e., extracted from English norms), and the resulting composite does not provide additional information beyond language exposure. Using such a composite measure to model data from language pairs in which reliable lexical frequency estimates would provide a more complete picture of the interplay between the effect of cognateness, lexical frequency, and degree of language exposure, on word bilingual acquisition.

It might be argued that our results reflect the fact that cognate translation equivalents are represented in the initial bilingual lexicon as the *same* lexical entry. Because cognates correspond to similar sounding word forms in equivalent referential contexts (e.g., hearing /ˈgat/ and /ˈga.to/ in the same situations), it is possible that infants classify both are as acceptable variations of the same word form, therefore treating them as a single lexical item. This would lead to a faster increase in cumulative learning instances, and to an earlier age‐of‐acquisition for cognate translation equivalents (for which listening to each word form contributes to the acquisition of its shared representation), compared to non‐cognates (for which listening to each word form contributes to the acquisition of a separate representation). This mechanism could potentially explain the earlier age‐of‐acquisition effect of cognates found in the present study, without the need of parallel activation playing any relevant role. Mitchell et al. (2023) discuss this possibility as a candidate explanation of the cognate facilitation effect, in which bilinguals only need to map one word form to the referent in the case of cognates, while mapping two distinct word forms in the case of non‐cognates. However, previous work on mispronunciation perception and learning of minimal pair words points in a different direction. Bilingual toddlers show monolingual‐level sensitivity to slight phonetic changes in a word form, according to their performance in word recognition tasks (Mani & Plunkett, 2011; Swingley, 2005; Tamási et al., 2017; Wewalaarachchi et al., 2017). The ability to differentiate between similar‐sounding word forms is also reflected in word learning, as bilinguals seem to be able to map minimal pairs to distinct referents (Mattock et al., 2010; Ramon‐Casas et al., 2017). Overall, it seems that bilinguals consider small differences in the phonological forms of words as relevant at the lexical level. We argue that this shows evidence that bilingual toddlers likely form distinct lexical representations for even near‐identical cognates.

Our study shares similar methodological limitations with previous work using vocabulary reports provided by caregivers. Such reports can be subject to measurement error induced by caregivers who may sometimes overestimate or underestimate participants' true probability of word acquisition (e.g., Houston‐Price et al., 2007). In the case of bilingual research additional biases may be in place. Although in the present study caregivers were explicitly instructed *not* to rely on their responses to Catalan words when responding to Spanish (and vice versa), it is possible that some caregivers assumed—at least to some extent—that because the child knew a word in one language, the child should also know the word in the other language. This bias would especially affect similar‐sounding words, i.e., cognates. Production estimates may be more sensitive to such biases, in part because of the slower pace at which infants' articulatory abilities develop, compared to their word recognition abilities (Hustad et al., 2021). This gap between comprehension and production is even larger in the less dominant language of bilingual children (Giguere & Hoff, 2022). For this reason, caregivers may be more uncertain about what words can be counted as *acquired* in this modality. Despite such potential biases, vocabulary checklist filled by caregivers show strong evidence of concurrent validity with other estimates of vocabulary size or lexical processing (Feldman et al., 2005).

The present study contributes with a specific data point to the complex landscape of bilingualism research. Bilinguals are a remarkably heterogeneous population difficult to be satisfactorily characterized in a comprehensive way (Sebastian‐Galles & Santolin, 2020). Bilinguals differ across multiple dimensions. Such differences span from exclusively linguistic factors; such as the amount of overlap between the phonemic inventories of the two languages being learned (e.g., low, like the case of English and Mandarin, or high, like the case of Spanish and Greek), to extralinguistic factors like the sociolinguistic situation in which the two languages co‐exist (e.g., in some regions both languages are co‐official and used in similar contexts, while in others, one of the languages has a smaller societal presence, i.e., heritage languages). This diversity of situations in which bilingual toddlers acquire language calls for special consideration of the generalisability of results in bilingualism research. Our sample, although homogeneous (e.g., similar parental educational level across participants), represents a particular bilingual sociolinguistic environment. As mentioned in the introduction, the languages involved in the present investigation, Catalan and Spanish, co‐exist as official languages and are known by the majority of the population. In addition, Catalan and Spanish are Romance languages and share a considerable amount of cognates. Extending our analyses to other bilingual populations learning typologically more distant languages, and whose languages tend to be used in more distinct contexts (e.g., heritage languages) should be a natural future step for the present investigation.

To conclude, our study provides novel insights about word acquisition in bilingual contexts and demonstrates how the presence of cognates in the children's linguistic input impacts the early formation of the lexicon. We found that during the acquisition of words from the lower exposure language, bilingual children seem to benefit more strongly from the word form's phonological similarity with its translation in the other language. Capitalizing on the notion of the *accumulator* of linguistic input, we put forward a theoretical account of bilingual word learning, in which cognateness interacts with language exposure to boost the acquisition of translation equivalents.

## CONFLICT OF INTEREST STATEMENT

The authors declare no conflicts of interest with regard to the funding source of this study.

## ETHICS STATEMENT

This study was conducted according to guidelines laid down in the Declaration of Helsinki and was approved by the Drug Research Ethical Committee (CEIm) of the IMIM Parc de Salut Mar, reference 2020/9080/I.

## Supporting information


Data S1.

## Data Availability

The data and code necessary to reproduce the analyses presented here are publicly accessible, as are the materials necessary to attempt to replicate the findings. The code is available at the following URL https://github.com/gongcastro/cognate‐beginnings. Materials are available at the following URL https://osf.io/hy984/. The analyses presented here were not preregistered.
