# Supplementary material for: Cognate beginnings to bilingual lexical acquisition
Source: Child Dev. 2024 Sep 23;96(1):286–300. doi: 10.1111/cdev.14170 (PMC11693823; doi:10.1111/cdev.14170)
Supplement: Supplementary file 1 — Data S1. [file CDEV-96-286-s001.docx]

Supplementary Materials

## SI1: Cross-linguistic lexical frequencies

The CHILDES database is commonly used to extract lexical frequencies from word-forms across many languages. The number of word-form for which information is available, and the number of individual children involved change substantially across corpora and languages. For instance, 134 corpora are available for English (excluding bilingual corpora) involving 1154 unique children in the relevant age rage (10 to 32 months) (see [Figure SI1](#sfig-participants-childes)), and providing information about the lexical frequency of 603,341 unique word-forms, based on 2,283,776. The number of corpora including Spanish tokens is lower than for English: 39 corpora include Spanish tokens, of which 15 are bilingual corpora. Nonetheless, these Spanish corpora (including the bilingual corpora) involve data from a sizable amount of participants, with 586 total distinct children included, and provide information about 35,577 unique word-forms in total, based on 312,407 tokens. Information available from the Catalan corpora is scarce. Only 8 corpora include Catalan tokens, of which 4 are bilingual corpora. Corpora including Catalan tokens (including bilingual corpora) involve 13 total distinct children, and provide information about 23,189 unique word-forms in total, based on 92,640 tokens. Given the low number of tokens, unique words, and critically, number of unique participants in the Catalan corpora, estimated lexical frequencies derived from Catalan corpora in CHILDES would be substantially unreliable.

|   Figure SI1. Number of distinct children included in the corpora of each language. |
| --- |

In line with the available literature, lexical frequency is an important predictor of word acquisition: word-forms with higher lexical frequency are acquired at earlier ages than word-forms with lower lexical frequency. For this reason, we included lexical frequency as a nuance predictor in our model, as this would provide more accurate estimations. Because of the scarcity of data about lexical information scores for Catalan words in CHILDES, we decided—in line with previous studies—to map English lexical frequencies onto Catalan and Spanish lexical frequencies, under the assumption that lexical frequency would, on average, remain stable across translation equivalents. While this approach is sub-optimal—lexical frequencies are expected to vary to some degree across languages—alternative strategies may induce more concerning artifacts.

First, using information from the Catalan CHILDES corpora may lead to unreliable estimates of lexical frequencies: the particular frequency with which a given word-form appears in a corpus may be due to the particular interaction taking place during the recording of the session (e.g., the word *bike* may appear several times if a bike was present in the room during the recording), or might reflect the particular interests of the child. The influence of both factors may be overweighted by the inclusion of recordings from a larger pool of children, as it is the case of the English corpora. But this is not the case for Catalan. Second, not using information about the lexical frequency of the items, even if mapped from the English CHILDES corpora, may reduce the accuracy of the estimations of the model, as lexical frequency is one of the strongest predictors of word acquisition. For these reasons, we made the methodological decision to impute the lexical frequencies extracted from the English corpora in CHILDES into the Catalan and Spanish translation equivalents.

Below, we report an estimation of the similarity between the lexical frequencies of several pairs of languages, as extracted from adult corpora. These frequencies were extracted from the PHOR database (Costa et al., 2023), which includes the following languages: American English (SUTBLEX-US, Brysbaert & New, 2009), British English (SUTBLEX-UK, Van Heuven et al., 2014), European Portuguese (SUBTLEX-PT, Soares et al., 2015), German (SUBTLEX-DE, Brysbaert et al., 2011), and Spanish (ESPAL, Duchon et al., 2013).

[Figure SI2](#sfig-corr) shows a scatter plot and univariate fitted linear model for each pair of languages, along with the Pearson correlation ($r$) between the lexical frequencies (expressed as $log_{10}\left( \text{Counts}+1 \right)$ of each pair of languages, and their corresponding 95% confidence interval and coefficient of determination ($R^{2}$).

|   Figure SI2. Pairwise scatter plots between adult lexical frequencies in English (UK and US), European Portuguese (PT), German (DE), and European Spanish (ES). |
| --- |

## SI2: model details

### Model structure and priors

Equation 1 shows a formal description of the model. We used Stan (Carpenter et al., 2017) as the probabilistic language behind the estimation of our Bayesian models in this study, with brms as its R interface (Bürkner, 2017). This language implements the Markov Chain Monte Carlo (MCMC) algorithm using the Hamiltonian Monte Carlo method (HMC) to explore the posterior distribution of the model. Broadly, this algorithm is used to iteratively sample the joint sampling space of the parameters to be estimated in the model, and compute, for each value sampled, its likelihood under some probability distribution previously defined. We run four MCMC chains, each 1,000 iterations long each.


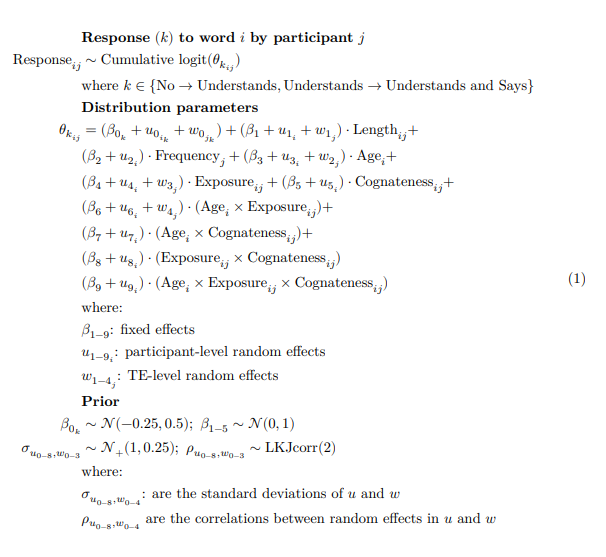


### Considerations on statistical power and sample size

There is little consensus about what approach is adequate for calculating the statistical power of a complex Bayesian model like the one in the present study, for several reasons. A first pitfall, shared with frequentist analysis, is that a closed solution for statistical power calculation is not possible or cannot be computed within reasonable time constraints. This rules out the use of many available pieces of software that are commonly offered for power analysis, as they commonly only consider the case of simpler models like t-tests, ANOVA, Pearson correlation, or regression (with only fixed effects), or trivial derivations of thereof. The more complicated case of multilevel models is usually not covered, not to mention those with a Bayesian approach.

An alternative way of estimating the statistical power of statistical test is simulation. This consists on simulating multiple datasets in which the hypothesised effect size is present, and fitting multiple instances of the model. The statistical power is derived from the proportion of contrasts that result in the rejection of the null hypothesis across datasets. Although this approach permits the estimation of statistical power in the case of more complex models, it involves costly computations. In the case of Bayesian models, and particularly the one in the present study, such cost can be infeasible. Sampling the posterior of our model took approximately seven days. Running this model, or an equivalent one, across 100 datasets (100 may even be considered too few by many) would take more than a year.

Following J. Kruschke (2014), we considered the precision of our estimates as a proxy to statistical power. In particular, we compared the width of the 95% HDI of the critical regression coefficient (*Exposure* $\times$ *Cognateness*) against some nominal interval width. We decided to use the half the width of the ROPE in the logit scale [-0.025, +0.025], that is, 0.05 as the reference interval width. The width of the fixed regression coefficient of *Exposure* $\times$ *Cognateness* ($\beta$ = -0.014, 95% HDI = [-0.017, -0.011]) was 0.006, around 8.933 times narrower than the reference interval. This indicates that the precision of the posterior 95% HDI of the critical parameter in the model is larger than required.

### Model diagnostics

One way to diagnose the behaviour of HMC is to inspect whether the different MCMC chains (if more than one) have converged to a similar region of the posterior. The Gelman-Rubin diagnostic ($\hat{R}$ or R-hat Gelman & Rubin, 1992) provides a measure of chain convergence by comparing the variance within each chain *versus* the variance between each chain. Both are expected to be identical when chains have perfectly converged, so that $\hat{R}=1$. Values lower than 1.01 are recommended, while values higher than 1.05 indicate that chains might have trouble converging and therefore the estimated parameters must be taken with caution. [Figure SI3](#sfig-rhats-neffs) (A) shows the distribution of $\hat{R}$ values for the coefficients of the fixed effect of our models, which we used for statistical inference. Most values are lower than 1.01, and never higher than 1.05, which provides evidence of successful MCMC convergence.

Another diagnostic of good MCMC converge is the ratio of effective sample size to total sample size (${N_{eff}}/N$), which indicates the proportion of samples in the chain that resulted from a non-divergent transition. Values closer to 1 are ideal, as they indicate that all posterior samples from the MCMC were used to estimate the posterior distribution of the parameter. Values larger than 0.1 are recommended. [Figure SI3](#sfig-rhats-neffs) (B) shows the distribution of the effective sample sizes of the coefficients of the fixed effects in our models. Most values are larger than 0.1, although model 0 ($M_{0}$) accumulates most effective sample sizes close to 0.1.

| 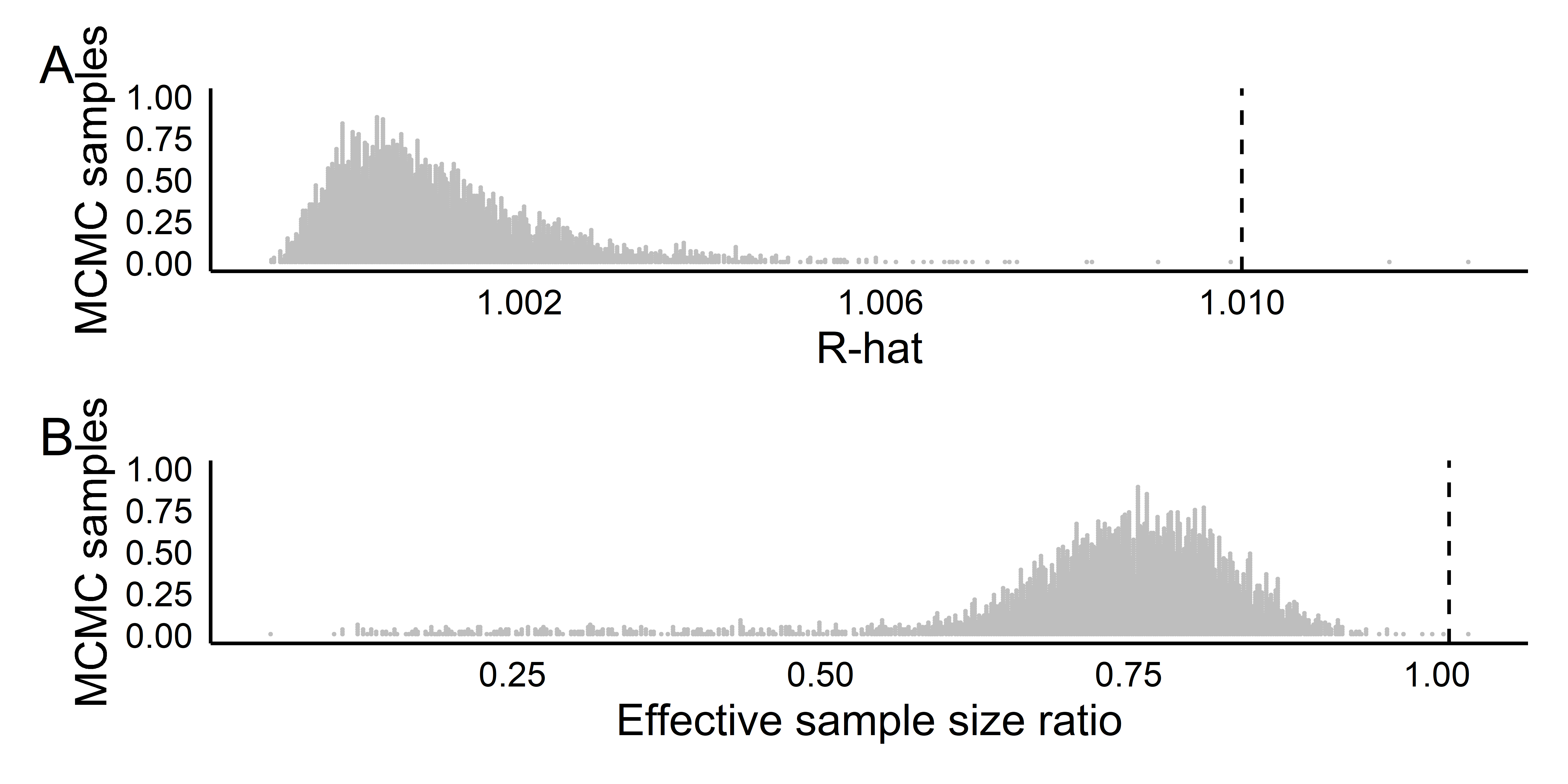  Figure SI3. MCMC convergence diagnostic of all parameters in the model. Each dot represents the score of one parameter. (A) Distribution of the Gelman-Rubin (R-hat) scores. (B) Distribution of the ratio of effective sample size. |
| --- |

Another way of assessing the behaviour of the HMC algorithm is to visualise the joint posterior distribution for pairs of parameters using bi-variate scatter plots. In [Figure SI4](#sfig-model-pairs) we show the pair-wise distribution of posterior samples. Broadly, posterior samples of two parameters should not be correlated. This is the case for all pairs of parameters but for the two intercepts. This is expected behaviour, given that these two parameters correspond to the thresholds between categories in the ordinal regression model, and the distance between both thresholds is fixed in the particular parametrisation of the model.

|   Figure SI4. Marginal distribution and bi-variate scatterplot of posterior samples for the fixed regression coefficients in Model 3. |
| --- |

Finally, we also assessed the predictive performance of the model by doing posterior-predictive checks (PPCs). this involves simulating new datasets from the model and the posterior distribution of its parameters, and checking that, overall, the distribution of the response variable across the simulated datasets is equivalent to the one in the observed dataset. [Figure SI5](#sfig-ppc) shows the PPCs of the model.

| 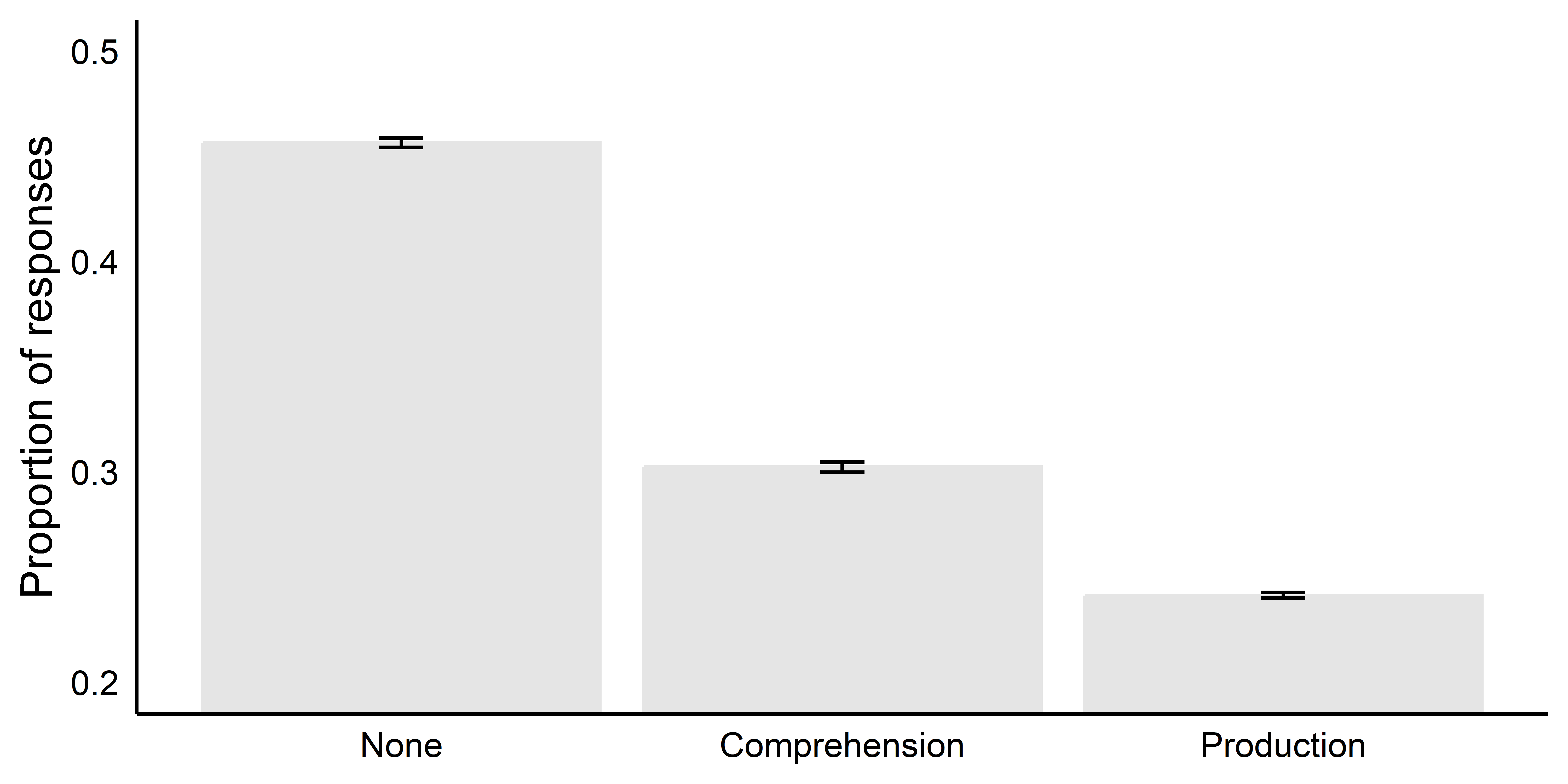  Figure SI5. Model posterior predictive checks (PPC). Bars indicate the observed proportion of responses to each category (No, Understands, and Understands and Says). Error bars represent the mean proportion of responses simulated from the posterior for each category, and its 95% interval. |
| --- |

## SI3: syllable frequency analysis

We define syllable frequency as the rate of appearance of individual syllables in the word-forms included in the [QUESTIONNAIRE NAME REDACTED FOR BLIND REVIEW] [REFERENCE REDACTED FOR BLIND REVIEW]. Each item corresponds to a Catalan or Spanish word, and has an associated phonological transcription in X-SAMPA format (Wells, 1995). These transcriptions are syllabified. Some examples:

Table SI1. Sample of items included in the BVQ questionnaire and their syllabified SAMPA transcriptions in Catalan and Spanish

| **Translation** | **Item** | **X-SAMPA** | **Syllables** | **Item** | **X-SAMPA** | **Syllables** |
| --- | --- | --- | --- | --- | --- | --- |
| grandma | àvia | “a.Bi.@ | 3 | abuela (yaya) | a”Bwe.la | 3 |
| sled | tobogan | tu.Bu”Gan | 3 | tobogán | to.Bo”Gan | 3 |
| clown | pallasso | p@“La.su | 3 | payaso | pa”j.so | 3 |
| water (beverage) | aigua | “aj.Gw@ | 2 | agua | “a.Gwa | 2 |
| pizza | pizza | “pid.z@ | 2 | pizza | “pid.sa | 2 |
| oven | forn | forn | 1 | horno | “o4.no | 2 |
| ball | pilota | pi”5O.t@ | 3 | pelota | pe”lo.ta | 3 |
| rake (object) | rastell | r@s“teL | 2 | rastrillo | ras”t4i.Lo | 3 |
| music | música | “mu.zi.k@ | 3 | música | “mu.si.ka | 3 |
| orange (food) | taronja | “t4OJ.Z@ | 2 | naranja | na”4an.xa | 3 |
| moon | lluna | “Lu.n@ | 2 | luna | “lu.na | 2 |
| head | cap | kap | 1 | cabeza | ka”be.Ta | 3 |
| neck | coll | kOL | 1 | cuello | “kue.Lo | 2 |
| door | porta | “pOr.t@ | 2 | puerta | “pwe4.ta | 2 |
| hammer | martell | m@r“teL | 2 | martillo | ma4”ti.Lo | 3 |

Most Catalan and Spanish words had two syllables, with Spanish words having three and four syllables more often than Catalan words. Less than 1% of the words included in the analyses presented in the main body of the manuscripts had five syllables. No words had more than five syllables (see [Figure SI6](#sfig-syll-number)). We extracted lexical frequencies from the English corpora in the CHILDES database (MacWhinney, 2000; Sanchez et al., 2019). Using the Catalan and Spanish corpora was not possible due to the low number of children and tokens included in the corpora.

| 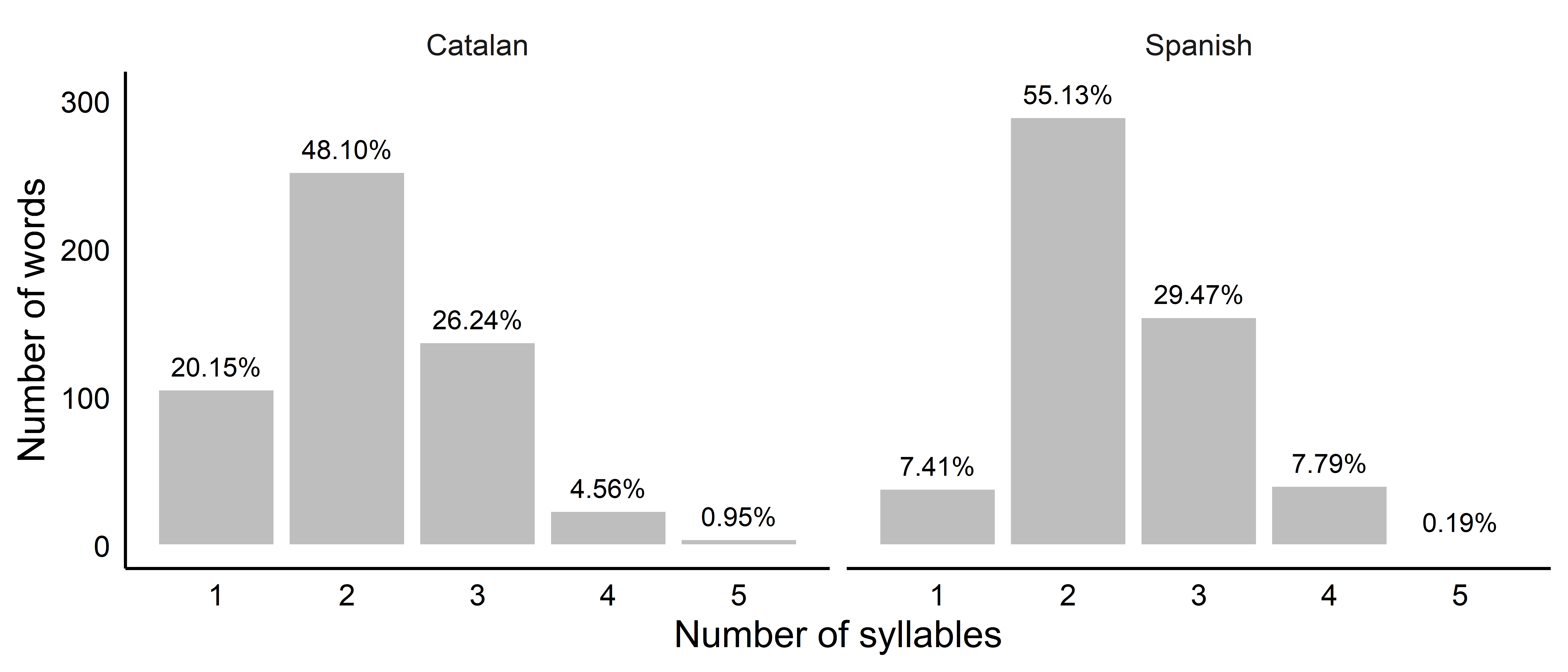  Figure SI6. Distribution of the number of syllables in Catalan and Spanish |
| --- |

We now present how syllable frequencies were calculated. Every exposure to a word-form also counts as an exposure to each of the syllables that make up such word. Every time a child hears the word *casa* [house], they are exposed to the syllables *ca* and *sa*. Syllables that appear embedded in words with higher lexical frequency will also be more frequent. To compute the relative frequency of each syllable in Catalan and Spanish (i.e., how many times the syllables appears in every million words in Catalan or Spanish speech), we summed the relative lexical frequency in CHILDES of every word that contains such syllable in the corresponding language. [Figure SI7](#sfig-syll-freq) shows the distribution of frequencies across syllables in Catalan and Spanish. In the log10 scale, syllable frequencies in Catalan and Spanish followed a slightly asymmetric distribution, with most syllables scoring around 1,000 counts per million, and a longer tail to the right of the distribution.

| 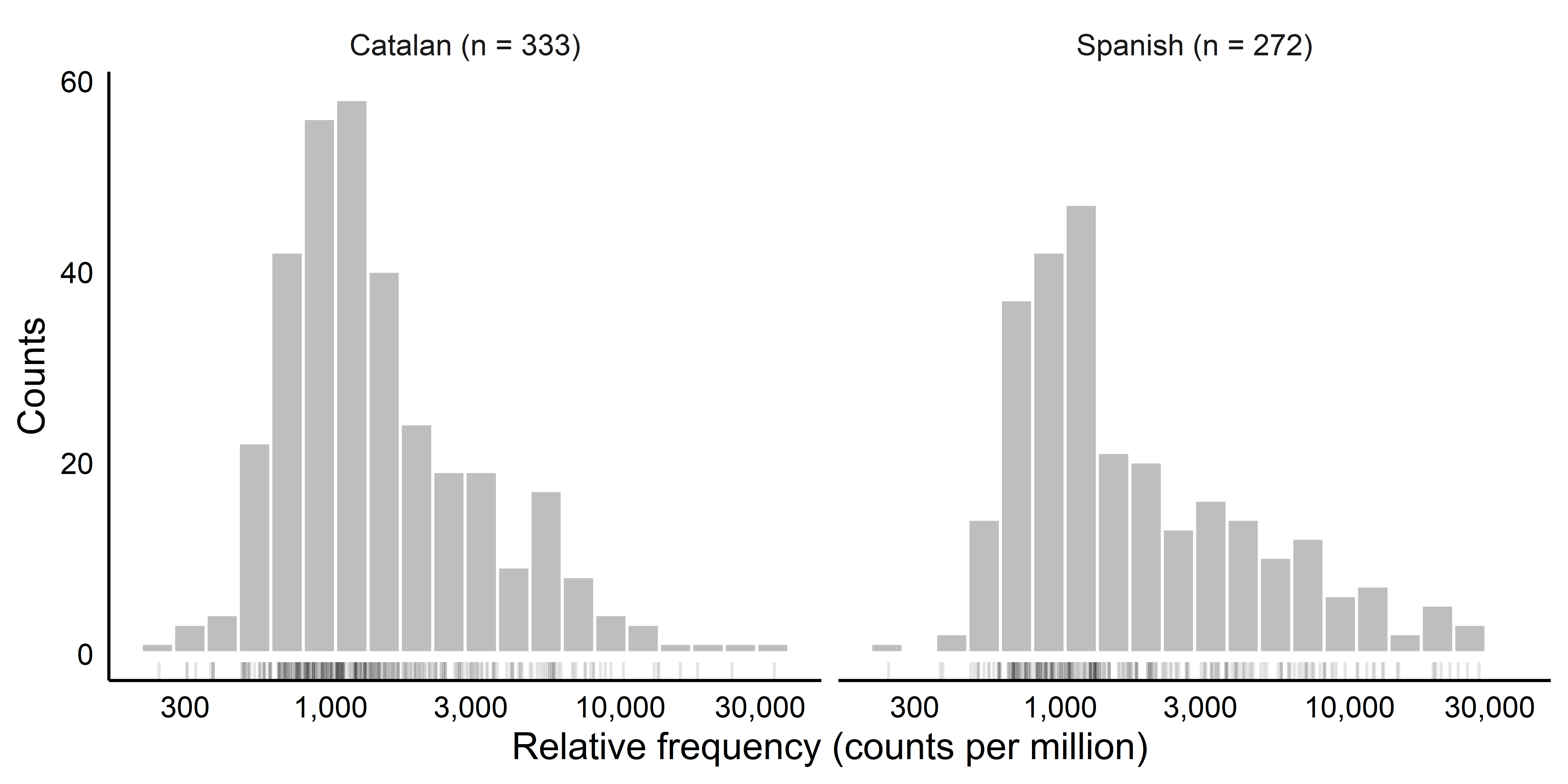  Figure SI7. Distribution of apositional syllable frequencies in Spanish and Catalan |
| --- |

To estimate the association between word-level syllabic frequency and cognateness, while controlling for the number of syllables in the word, as words are expected to necessarily increase the syllabic frequency of the word, we fit a multilevel, Bayesian linear regression model with syllabic frequency (the sum of the syllabic frequency of the syllables in a word) as response variable, and the main effect of the number of syllables (*Syllables*) and *Cognateness* (Levenshtein similarity between a word and its translation equivalent, Levenshtein, 1966) as predictors. We added translation equivalent-level random effects for the intercept and the main effect of *Syllables* (some translation pairs had a different number of syllables in each language). We used a Gaussian distribution to model syllabic frequency scores after standardising this variable and the predictors. We used a weakly informative prior for all parameters involved in the model (see Equation 2 for a formal equation of this model and its prior). We conducted statistical inference by evaluating the proportion of the 95% highest density interval (HDI) of the posterior posterior distribution of each coefficient that fell into the region of practical equivalence (ROPE, see the main manuscript for a more detailed explanation, J. K. Kruschke & Liddell, 2018).


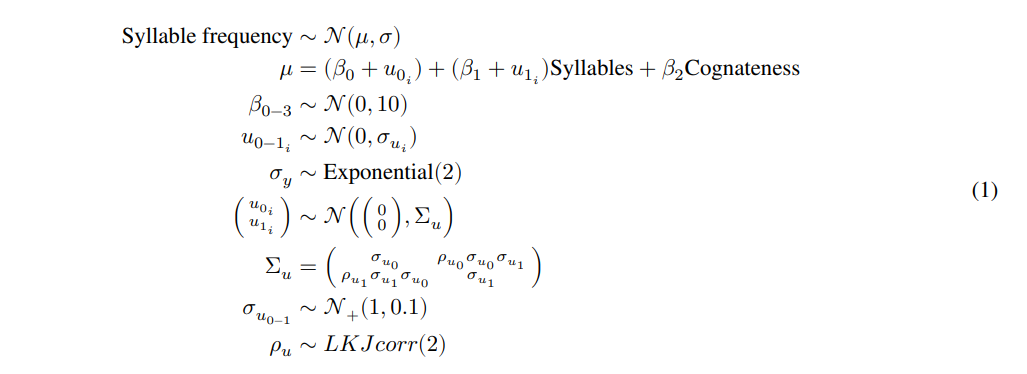


We fit this model running 4 sampling chains with 1,000 iterations each. Table SI2 shows a summary of the posterior distribution of the fixed effects in the model. As expected, words with more syllables scored higher in syllabic frequency: all posterior draws for the regression coefficient of the main effect of this predictor fell outside the ROPE defined between -0.5 and +0.5 ($\beta$ = 5.64, 95% HDI = [5.58, 5.71]). Keeping the number of syllables constant, the effect of cognateness was negligible: all of the posterior distributions of this predictor fell within the ROPE, providing evidence that the true value of the increment in syllabic frequency for every increase in cognateness is equivalent to zero ($\beta$ = 0.01, 95% HDI = [-0.06, 0.07]).

Posterior distribution of regression coefficients. β: median of the posterior distribution in the probability scale. 95% HDI: 95% highest density interval of the distribution. *p*(ROPE): overlap between the 95% HDI and the ROPE, indicating the posterior probability that the true value of the coefficient is equivalent to zero.

|  | ***β*** | **95% HDI** | ***p*(ROPE)** |
| --- | --- | --- | --- |
| Intercept | 16.088 | [16.023, 16.163] |  |
| Syllables (+1 SD, 0.802) | 5.643 | [5.579, 5.713] | 0.000 |
| Cognateness (+1 SD, 0.24) | 0.009 | [-0.063, 0.073] | 1.000 |

[Figure SI8](#sfig-syll-marginal) shows the median posterior-predicted syllabic frequencies for words with one to four syllables, for the whole range of cognateness values. Overall, cognate words’ syllabic frequency is equivalent to that of non-cognates. This suggests that the cognate facilitation effect in word acquisition reported in the present study is not the result from an association between cognateness and higher syllabic frequencies.

| 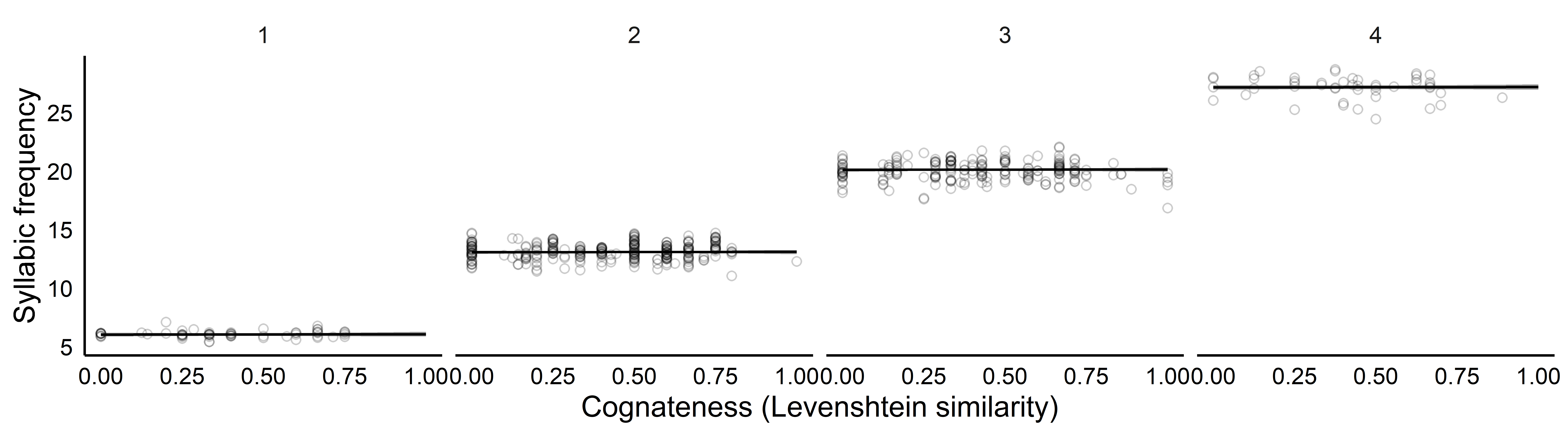  Figure SI8. Posterior predictions of the syllabic frequency model. Thicker lines indicate the median of the posterior predictions, and thinner lines indicate individual posterior predictions. |
| --- |

## SI4: Language exposure-weighted lexical frequency predictor

We developed the *Exposure* predictor to account for the fact that bilinguals’ exposure to a given word-form is not only a function of the word-form’s lexical frequency, but also of the quantitative input they receive from the language such word-form belongs to. We expressed lexical frequencies as the product between both variables. As in the main model, we first extracted the child-directed lexical frequency of each word-form from the English corpora of the CHILDES database (MacWhinney, 2000), and mapped them into their Catalan and Spanish translation equivalents. We then transformed the resulting lexical frequencies into Zipf scores. To obtain the new composite measure, which we term the language-exposure weighted lexical frequency (LEWF), we multiplied the resulting lexical frequencies by the child’s degree of exposure (DoE) to Catalan or Spanish (whichever language the word-form belongs to) (see Equation 1).


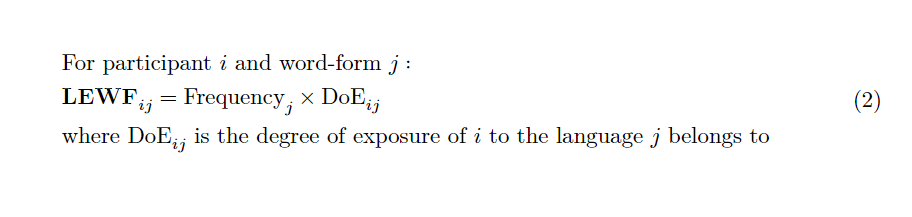


For instance, for a child whose degree of exposure is 80% for Catalan and 20% for Spanish, the expected *Exposure* score to the Catalan word-form *cotxe* [*car*]—with a lexical frequency of 6.33—would be 5.06, while that of its translation to Spanish *coche* would be 1.27.

[Figure SI9](#sfig-coefs-composite) shows the posterior distribution of the fixed regression coefficients of the model that included *LEWF* as a fixed effect, in interaction with *Age* and *Cognateness*, and which did not include *Frequency* as a co-variate (as this variable is now included in the *LEWF* predictor). Overall, results are equivalent, indicating that *LEWF* might be a useful estimate of lexical frequency in bilinguals.

| 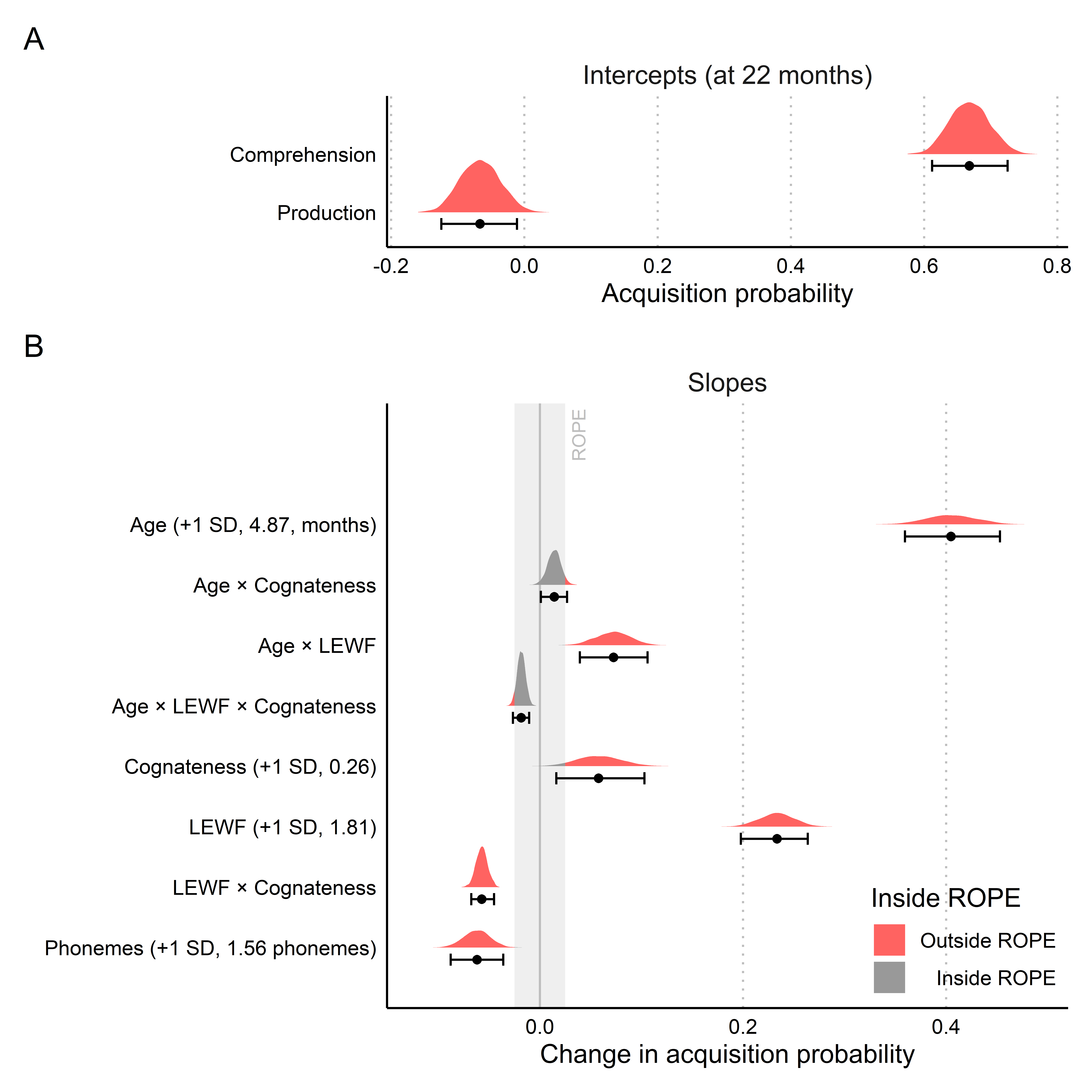  Figure SI9. Posterior distribution of fixed regression coefficients. Values have been transformed to the probability scale for interpretability. (A) Posterior distribution of intercepts for *Comprehension* and for *Comprehension and Production*. The X-axis shows the estimated average probability of acquisition when all predictors are set at zero. The grey rectangle shows the region of practical equivalence (ROPE, [-0.025, +0.025]). (B) Posterior distribution of slopes. The X-axis shows the estimated average change in probability of acquisition associated with a change of one standard deviation in the predictor, when the other predictors are set at zero. |
| --- |

## References

Brysbaert, M., Buchmeier, M., Conrad, M., Jacobs, A. M., Bölte, J., & Böhl, A. (2011). The word frequency effect. *Experimental Psychology*.

Brysbaert, M., & New, B. (2009). Moving beyond kučera and francis: A critical evaluation of current word frequency norms and the introduction of a new and improved word frequency measure for american english. *Behavior Research Methods*, *41*(4), 977–990.

Bürkner, P.-C. (2017). Brms: An r package for bayesian multilevel models using stan. *Journal of Statistical Software*, *80*, 1–28. https://doi.org/10.18637/jss.v080.i01

Carpenter, B., Gelman, A., Hoffman, M. D., Lee, D., Goodrich, B., Betancourt, M., Brubaker, M., Guo, J., Li, P., & Riddell, A. (2017). Stan: A probabilistic programming language. *Journal of Statistical Software*, *76*(1). https://doi.org/10.18637/jss.v076.i01

Costa, A. S., Comesaña, M., & Soares, A. P. (2023). PHOR-in-one: A multilingual lexical database with PHonological, ORthographic and PHonographic word similarity estimates in four languages. *Behavior Research Methods*, *55*(7), 3699–3725.

Duchon, A., Perea, M., Sebastián-Gallés, N., Martı́, A., & Carreiras, M. (2013). EsPal: One-stop shopping for spanish word properties. *Behavior Research Methods*, *45*, 1246–1258.

Gelman, A., & Rubin, D. B. (1992). Inference from iterative simulation using multiple sequences. *Statistical Science*, *7*(4), 457–472. https://www.jstor.org/stable/2246093

Kruschke, J. (2014). *Doing bayesian data analysis: A tutorial with r, JAGS, and stan*. Academic Press.

Kruschke, J. K., & Liddell, T. M. (2018). The bayesian new statistics: Hypothesis testing, estimation, meta-analysis, and planning from a bayesian perspective. *Psychonomic Bulletin &Review*, *25*, 178–206. https://doi.org/10.3758/s13423-016-1221-4

Levenshtein, V. I. (1966). Binary codes capable of correcting deletions, insertions, and reversals. *Soviet Physics-Doklady*, *10*, 707–710.

MacWhinney, B. (2000). *The CHILDES project: The database* (Vol. 2). Psychology Press.

Sanchez, A., Meylan, S. C., Braginsky, M., MacDonald, K. E., Yurovsky, D., & Frank, M. C. (2019). Childes-db: A flexible and reproducible interface to the child language data exchange system. *Behavior Research Methods*, *51*(4), 1928–1941.

Soares, A. P., Machado, J., Costa, A., Iriarte, Á., Simões, A., Almeida, J. J. de, Comesaña, M., & Perea, M. (2015). On the advantages of word frequency and contextual diversity measures extracted from subtitles: The case of portuguese. *Quarterly Journal of Experimental Psychology*, *68*(4), 680–696.

Van Heuven, W. J., Mandera, P., Keuleers, E., & Brysbaert, M. (2014). SUBTLEX-UK: A new and improved word frequency database for british english. *Quarterly Journal of Experimental Psychology*, *67*(6), 1176–1190.

Wells, J. C. (1995). *Computer-coding the IPA: A proposed extension of SAMPA*. *4*(28), 1995.
